# Evaluation of the reliability of large language models for ASA-PS classification in cardiovascular surgery: a pilot study

**DOI:** 10.1186/s40981-026-00858-4

**Published:** 2026-04-15

**Authors:** Keisuke Iwabu, Takashi Juri, Shogo Tsujikawa, Hideki Hino, Yusuke Funai, Koichi Suehiro, Takashi Mori

**Affiliations:** Department of Anesthesiology, Osaka Metropolitan University Graduate School of Medicine, 1-5-7 Asahi-Machi, Abeno-Ku, Osaka City, Osaka, 545-8585 Japan

**Keywords:** American Society of Anesthesiologists Physical Status, Cardiovascular Surgery, Large language models, Generative artificial intelligence, ChatGPT, Gemini

## Abstract

**Background:**

Large language models (LLMs) have shown promising performance for ASA Physical Status (ASA-PS) classification, but prior work suggests reduced agreement in high-risk patients. We evaluated LLM reliability for ASA-PS classification in cardiovascular surgery.

**Results:**

Thirty-two anonymized cases were rated by two residents, two board-certified cardiovascular anesthesiologists, and four LLM modes (ChatGPT: GPT-5.2 Instant and GPT-5.2 Thinking; Gemini: Gemini 3 Fast and Gemini 3 High Thinking); all LLM assessments were zero-shot. Overall agreement across evaluators was moderate (intraclass correlation coefficient [ICC] 0.49–0.52); agreement between each LLM and specialists was good (ICC 0.61–0.65). Exact-match to a five-specialist consensus was 42.2% for residents versus 59.4–75.0% for LLMs; classifications outside the range of ratings assigned by individual specialists were rare (0–3.1%).

**Conclusions:**

In cardiovascular surgery, contemporary LLMs showed good concordance with cardiovascular anesthesiologists and exceeded resident agreement with expert consensus, supporting prospective multicenter validation as adjuncts for ASA-PS assessment and training.

**Supplementary Information:**

The online version contains supplementary material available at 10.1186/s40981-026-00858-4.

## Background

The American Society of Anesthesiologists Physical Status (ASA-PS) classification system, initially introduced by Saklad et al. in 1941, remains the most widely utilized tool for preoperative risk assessment [1, 2]. The primary advantage of ASA-PS is its simplicity: the scale consists of six levels (I–VI), with an optional “E” suffix indicating emergency procedures. Nevertheless, this simplicity introduces subjectivity, and several studies have highlighted substantial inter-rater variability in ASA-PS assessments [3–5].

Inaccurate ASA-PS classification can result in serious consequences. Underestimation of ASA-PS has been shown to result in delays in critical and potentially life-saving interventions and is independently associated with increased 30-day postoperative mortality [5–7]. Conversely, overestimation may result in unnecessary preoperative testing, inflated healthcare costs, and inappropriate allocation of limited intensive care resources [5, 8]. Therefore, ensuring accurate and consistent ASA-PS classification is essential for patient safety and the efficient use of healthcare resources.

In parallel, rapid advances in artificial intelligence (AI) have accelerated interest in the clinical applicability of large language models (LLMs), including ChatGPT (OpenAI) and Gemini (Google), across a range of medical tasks [9–13]. A recent prospective multicenter study demonstrated high concordance between ASA-PS assessments made by anesthesiologists and GPT-4 [10]. However, the study also reported decreased accuracy in cases classified as ASA-PS IV, suggesting diminished model performance in patients with extensive or complex comorbidities. Patients undergoing major cardiovascular surgery are commonly classified as ASA-PS III or IV due to significant underlying conditions, including advanced valvular heart diseases or severely reduced left ventricular function associated with coronary artery disease [14]. Recent advancements in LLM technology suggest the possibility that ChatGPT and Gemini could accurately classify ASA-PS even in such high-risk patient populations. If validated, these models may serve as an adjunctive tool to support ASA-PS assessments by anesthesiologists or to aid training for anesthesia residents.

In this pilot study, we evaluated the agreement between ASA-PS classifications generated by ChatGPT and Gemini and those assigned by anesthesiologists for patients undergoing cardiovascular surgery.

## Methods

### Study design and participants

Ethical approval for this retrospective, single-center, cross-sectional pilot study was obtained from the Institutional Review Board of Osaka Metropolitan University (Approval No. 2024–120). All procedures were conducted in accordance with the ethical standards of the 1964 Helsinki Declaration and its later amendments. As all personal identifiers were removed before analysis, the requirement for written informed consent was waived. We included patients who underwent cardiovascular surgery under general anesthesia at Osaka Metropolitan University Hospital between April 2020 and May 2024. Rare cases in which the disease diagnosis or planned surgical procedure occurred in five or fewer patients during the study period were excluded. All remaining eligible cases were enumerated and assigned unique study IDs. We then selected 32 cases by simple random sampling without replacement using a computer-generated random sequence.

### Data collection and anonymization

An anonymized dataset was constructed from the 32 selected cases. For each case, preoperative information was extracted from the electronic medical records. This information included demographics (e.g., age, sex, body mass index), as well as findings from physical examinations, laboratory tests, physiological tests, and imaging reports. A comprehensive summary of these data is presented in Table 1. To standardize the case summaries, we used a prespecified extraction rule. Each summary routinely included age, sex, body mass index, NYHA functional class, planned procedure, present illness, social history (smoking status), past medical history/comorbidities, key laboratory values (hemoglobin, platelet count, and eGFR), and ECG findings; other test results (e.g., additional laboratory parameters, echocardiography including TEE, CT, and pulmonary function tests) were included only when abnormal. To ensure anonymity, references in the medical history that could reveal specific dates were removed. Furthermore, comorbidities that were considered sufficiently rare—specifically those encountered in three or fewer cases by either of the two cardiovascular anesthesia specialists over the course of 1 year—were excluded to minimize the risk of patient identification. Other potentially identifiable data elements were also excluded. Examples of anonymized case data are provided in the supplemental material (Supplemental Material 1).

**Table 1:** Dataset

Category	Variables
Demographics	Age, Sex, Diagnosis, Planned surgery, History of Present Illness, Past Medical History, Smoking habits
Physical Examinations	Height, Weight, Body Mass Index, New York Heart Association (NYHA) Heart Failure Classification
Laboratory test results	Serum Creatinine level, estimated Glomerular Filtration Rate, etc
Physiological test results	Electrocardiography, Pulmonary function test result
Imaging reports	Chest radiography, Computed Tomography

### ASA-PS classification procedures

ASA-PS classifications were independently assigned to each of the 32 anonymized cases using two distinct methods: (1) evaluation using LLMs and (2) independent assessments by anesthesiologists. For the LLM–based evaluation, four different modes were used: GPT-5.2 Instant, GPT-5.2 Thinking, Gemini 3 Fast, and Gemini 3 High Thinking.

All AI-based assessments were conducted using a zero-shot approach (no examples, no rubric, and no iterative prompt refinement). We used a fixed prompt (“I will now present 32 cases. Please evaluate the ASA-PS classification for each case.”) and presented each case as a comprehensive summary one at a time sequentially within a single session for each model. Each model was queried only once per case, and no repeated runs were performed. From each response, we recorded only the ASA-PS label; any accompanying rationale was disregarded. All model outputs provided a single ASA-PS class per case. The fixed prompt and an example LLM interaction (case 1), including the model response, are provided in Supplemental Material 2. All LLM evaluations were performed via the web-based user interfaces using default settings on January 7, 2026. No additional system/developer instructions, external tools, or iterative prompt refinement were used beyond the fixed user prompt and summaries. Generation parameters (e.g., temperature) were not available to the user and could not be verified in the interface at the time of evaluation.

Independent assessments were performed by four raters, including two anesthesia residents and two board-certified cardiovascular anesthesiologists. Both residents had six months of full-time clinical experience in anesthesiology at the time of evaluation. Each rater received the same anonymized case summaries as the LLMs and had no access to additional clinical information beyond these summaries. No reference materials, including the ASA-PS classification table or related guidelines, were provided or consulted during the assessments. Each rater evaluated all 32 cases. The sequence of cases was identical across all raters to minimize order-related bias.

In addition, a panel of five cardiovascular anesthesiology specialists (including the two board-certified specialists from the independent assessment group) collaboratively reviewed the same 32 cases. This panel determined consensus-based ASA-PS classifications (termed "consensus scores") for each patient. These consensus scores served as reference classifications for comparison against both LLMs-generated and resident-assigned ASA-PS ratings.

### Outcome measures

The primary outcome measure was the agreement between ASA-PS classifications generated by LLMs and those independently assigned by anesthesiologists (residents and board-certified specialists). Agreement was quantitatively assessed using intraclass correlation coefficients (ICC). Additionally, we evaluated the alignment of LLMs-derived ratings and resident-assigned ratings with the consensus scores established by the specialist panel.

### Statistical analysis

Statistical analyses were performed using MedCalc software (version 23.0.2.; MedCalc Software Ltd., Ostend, Belgium). Inter-rater reliability between LLM-generated and anesthesiologist-assigned ASA-PS classifications was assessed by calculating ICC employing a two-way mixed-effects model for absolute agreement (ICC (3,1)). For analyses, the “E” suffix was removed, and ASA-PS was analyzed using the numeric class only (I-IV) (e.g., “II E” was treated as “II”). ICC values were interpreted based on standard guidelines: values < 0.40 indicated poor agreement; 0.40 to 0.59, moderate agreement; 0.60 to 0.74, good agreement; and ≥ 0.75, excellent agreement [15]. Descriptive statistics were also generated to compare the distribution of ASA-PS classification assigned by LLM and residents, relative to the consensus scores.

In addition, pairwise quadratic weighted kappa coefficients were calculated between each LLM and the human raters (board-certified cardiovascular anesthesiologists and residents). Weighted kappa values were interpreted according to established guidelines: ≤ 0.20, slight; 0.21 to 0.40, fair; 0.41 to 0.60, moderate; 0.61 to 0.80, substantial; and 0.81 to 1.00, almost perfect agreement [16].

### Sample size calculation

The priori sample size was determined based on the desired precision of the ICC estimate. Assuming a total of five raters (four human anesthesiologists plus LLM) and an expected ICC of 0.60 with a confidence interval width of 0.30, as reported in a previous study [17], we calculated that 32 cases would be required to achieve a significance level of 5% (α = 0.05) [18]. Accordingly, the study was designed with 32 cases to ensure adequate statistical power for estimating inter-rater reliability.

## Results

### Distribution of ASA-PS classifications across evaluator groups

**Fig. 1:**
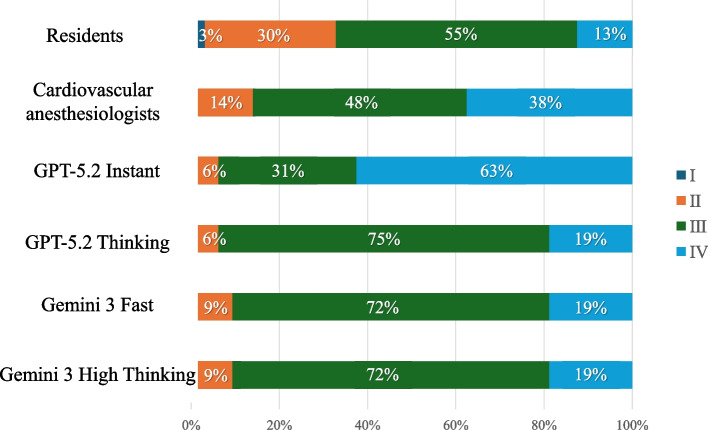
Distribution of ASA-PS Classifications Assigned by Residents, Cardiovascular Anesthesiologists, and generative artificial intelligence. The figure shows the percentage distribution of ASA-PS classifications assigned to 32 cardiovascular surgery cases by six groups: anesthesiology residents, board-certified cardiovascular anesthesiologists, and generative artificial intelligence using GPT-5.2 Instant, GPT-5.2 Thinking, Gemini 3 Fast, and Gemini 3 High Thinking

Figure 1summarizes the distribution of ASA-PS classifications assigned by anesthesiology residents, board-certified cardiovascular anesthesiologists, and large language models (GPT-5.2 Instant/Thinking and Gemini 3 Fast/High Thinking). Residents most frequently assigned ASA-PS III (55%), followed by ASA-PS II (30%), ASA-PS IV (13%), and ASA-PS I (3%). Cardiovascular anesthesiologists similarly concentrated ratings in higher-risk categories, with 48% classified as ASA-PS III and 38% as ASA-PS IV, while 14% were ASA-PS II. Across the LLM settings, assignments were overwhelmingly ASA-PS III–IV: GPT-5.2 Instant favored ASA-PS IV (63%) over ASA-PS III (31%) and ASA-PS II (6%), whereas GPT-5.2 Thinking primarily assigned ASA-PS III (75%), with fewer ASA-PS IV (19%) and ASA-PS II (6%). Gemini 3 Fast and Gemini 3 High Thinking exhibited near-identical distributions, assigning most cases to ASA-PS III (72%), with the remainder classified as ASA-PS IV (19%) and ASA-PS II (9%). Overall, the LLM outputs mirrored cardiovascular anesthesiologists in emphasizing ASA-PS III–IV classifications, although the relative weighting of ASA-PS III versus IV varied by model variant.

### Inter-rater agreement among evaluators

Overall inter-rater agreement in ASA-PS classification across the 32 cases was comparable across all evaluated LLM conditions when evaluated together with the four human raters (two anesthesiology residents and two board-certified cardiovascular anesthesiologists). The overall ICC was 0.49 (95% CI: 0.33–0.66) when incorporating GPT-5.2 Instant, 0.50 (95% CI: 0.34–0.67) with GPT-5.2 Thinking, 0.52 (95% CI: 0.36–0.68) with Gemini 3 Fast, and 0.52 (95% CI: 0.36–0.68) with Gemini 3 High Thinking; all values indicated moderate agreement.

When the analysis was restricted to agreement with the two board-certified cardiovascular anesthesiologists, ICCs indicated good agreement for GPT-5.2 Instant, GPT-5.2 Thinking, Gemini 3 Fast, and Gemini 3 High Thinking (ICC = 0.65 [95% CI: 0.47–0.80], 0.61 [0.42–0.77], 0.62 [0.43–0.78], and 0.62 [0.43–0.78], respectively).

Supplementary pairwise quadratic weighted kappa analysis yielded concordant findings. Weighted kappa values between LLMs and the two board-certified cardiovascular anesthesiologists ranged from 0.46 to 0.74 (moderate to substantial agreement), while values between LLMs and residents showed greater variability, ranging from 0.14 to 0.73 (slight to substantial agreement) (Supplemental Table 1).

### Agreement with expert consensus

To further assess classification accuracy, we calculated the percentage of exact matches with the consensus ASA-PS scores determined by a panel of five board-certified cardiovascular anesthesiologists. The residents’ scores matched the consensus classification in only 42.2% of cases. In contrast, the LLMs demonstrated higher concordance with the expert consensus: GPT-5.2 Instant achieved exact matches in 75.0% of cases, GPT-5.2 Thinking in 68.8%, Gemini 3 Fast in 62.5%, and Gemini 3 High Thinking in 59.4% (Table 2). These findings indicate that, among the evaluated approaches, GPT-5.2 Instant showed the closest alignment with expert-level ASA-PS classification.

**Table 2:**
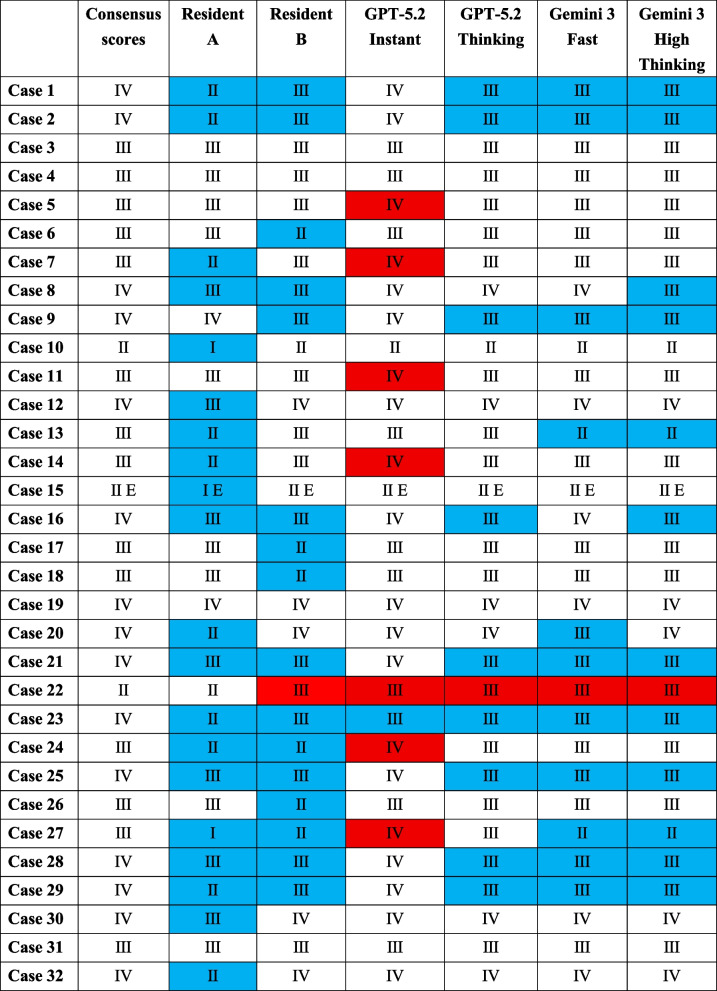
ASA-PS Classifications Assigned by Residents and LLMs Compared to Consensus Scores by Cardiovascular Anesthesiologists

Consensus scores represent the modal ASA-PS classification determined by a panel of five board-certified cardiovascular anesthesiologists. Cells in which the assigned score is lower than the consensus are highlighted in blue; those with higher scores are highlighted in red

In addition, classifications that did not correspond to any of the ASA-PS ratings assigned by the individual cardiovascular anesthesiologists contributing to the consensus were rare: 0% for GPT-5.2 Instant, 3.1% for GPT-5.2 Thinking, 0% for Gemini 3 Fast, and 0% for Gemini 3 High Thinking. Overall, these results suggest that ASA-PS classifications generated by the LLMs were broadly comparable to those made by cardiovascular anesthesiology experts.

## Discussion

Our pilot study evaluated the reliability of contemporary LLMs for ASA-PS classification in a cardiovascular surgical population in which high-risk profiles (predominantly ASA-PS III–IV) are common. Across 32 cases, overall inter-rater agreement remained moderate when each LLM was evaluated together with four human raters (two anesthesiology residents and two board-certified cardiovascular anesthesiologists), with similar ICCs across model variants (0.49–0.52). In contrast, when agreement was restricted to the two board-certified cardiovascular anesthesiologists, all LLMs demonstrated good agreement (ICC 0.61–0.65), with GPT-5.2 Instant showing the highest concordance (ICC 0.65). These findings indicate that LLM-generated ASA-PS classifications aligned more closely with expert-level judgment than would be expected if model behavior simply mirrored less-experienced clinician ratings.

When benchmarked against consensus scores determined by a five-specialist panel, LLMs also achieved higher exact-match rates than residents (42.2%), ranging from 59.4% (Gemini 3 High Thinking) to 75.0% (GPT-5.2 Instant). Because exact-match is a stringent criterion for an ordinal, multi-level scale, these concordance rates are clinically meaningful in the context of preoperative risk stratification, where documentation consistency and communication of baseline physiologic risk are essential. Importantly, classifications outside the range of ASA-PS ratings assigned by any of the individual cardiovascular anesthesiologists contributing to the consensus were rare (0–3.1%), suggesting that LLM outputs generally remained within the spectrum of plausible expert assessments rather than producing idiosyncratic ratings. Together, these results support the interpretation that, at least in this cohort, LLM-based ASA-PS classification can approximate specialist practice patterns.

Although the underlying reasons cannot be determined from this study design, two factors may have contributed to the higher agreement between LLMs and cardiovascular anesthesiologists. First, contemporary model families may capture complex comorbidity patterns more effectively than earlier generations, which could partially explain the improved alignment with expert ratings observed in this high-risk cardiovascular cohort. Second, our standardized case summaries prioritized clinically relevant abnormal findings from laboratory and imaging reports; this input format may have emphasized the same features that specialists typically attend to when assigning ASA-PS, thereby increasing concordance with expert assessments.

The distributional patterns across evaluator groups provide additional context. The LLM outputs, like those of the cardiovascular anesthesiologists, largely concentrated in ASA-PS III–IV; however, the relative balance between III and IV differed by model variant. In particular, GPT-5.2 Instant favored ASA-PS IV more than the other models, whereas GPT-5.2 Thinking and both Gemini modes classified most cases as ASA-PS III. While this study did not quantify directional error (over- vs under-classification) relative to the consensus, such distributional differences may influence downstream workflow (e.g., perceived ICU need or intensity of perioperative planning) and should be explicitly evaluated in future studies, particularly given the known clinical and resource implications of ASA-PS misclassification [5–8].

Our findings should also be considered in the context of previous research on AI-driven ASA-PS classification [9–11, 19, 20]. Prior studies have raised concerns regarding the accuracy of earlier AI models in classifying high-risk patients (ASA III–IV), often reporting either reduced accuracy or systematic underestimation in these groups. For example, a multicenter evaluation of an earlier model, GPT-4, demonstrated excellent overall concordance with human anesthesiologists (Cohen’s κ = 0.86) but showed notably reduced accuracy for ASA IV patients [10]. Similarly, another GPT-based evaluation highlighted a trend toward underestimation in patients classified at the higher end of the ASA scale (ASA IV–V) [11]. In the present study, which specifically targeted cardiovascular surgical patients who frequently fall into ASA-PS III–IV categories, agreement between LLMs and cardiovascular anesthesiologists was consistently good (ICC 0.61–0.65), and pairwise quadratic weighted kappa values between LLMs and specialists ranged from 0.46 to 0.74, indicating moderate to substantial agreement. Exact-match rates with the expert consensus were highest for GPT-5.2 Instant. Although cross-study comparisons must be made cautiously due to differences in case mix, prompts, model versions, and reference standards, our results are compatible with the possibility that newer model families may better handle complex comorbidity profiles typical of cardiovascular surgery, or that the presence of salient high-risk features in such cases provides clearer signals for classification.

In contrast, the lower agreement between residents and the specialist consensus is also clinically interpretable. In an exploratory review of discordant cases, we noted a recurring pattern in which the specialist panel assigned ASA-PS IV for patients with clearly advanced functional limitation (e.g., NYHA class $\ge$ III) and/or severe valvular disease (e.g., severe aortic stenosis), whereas residents sometimes assigned ASA-PS III for these same cases. This pattern may reflect limited anesthesiology experience with high-acuity cardiovascular pathology and less established calibration regarding how severe cardiac disease translates into global ASA-PS severity. Although we did not perform formal statistical analyses of discordance drives, Fig. 1 suggests that residents assigned ASA-PS II more frequently than specialists and LLMs, consistent with a tendency toward under-classification in this cohort. These observations highlight a potentially actionable educational gap and support future work that prospectively quantifies the direction and clinical determinants of resident-consensus discrepancies.

From a practical standpoint, these results suggest several potential applications. First, LLM outputs may serve as an adjunctive “second reader” to support ASA-PS assignment in settings where inter-rater variability is known to be substantial [3–5]. Second, the observed gap between resident exact-match rates and LLM exact-match rates raises the possibility of educational use: trainees could compare their ASA-PS assignments with model outputs and, under faculty supervision, review discordant cases to reinforce consistent interpretation of ASA-PS criteria.

However, some limitations of this pilot study should be acknowledged. First, the five-specialist consensus used in this study should be interpreted as a pragmatic reference comparator rather than a true gold standard. ASA-PS classification is inherently subjective, and even expert consensus reflects aggregated clinical judgment that may vary across institutions and practice cultures. Accordingly, our findings should be framed as agreement with expert consensus and agreement with specialist raters, not as definitive “accuracy” of any model. Second, our analysis was limited to 32 patients from a single institution, which may limit the generalizability of the findings to other clinical settings and patient populations. Validation in larger, multi-institutional cohorts is necessary to confirm broader applicability. Third, all LLM assessments were performed under a zero-shot condition. Furthermore, all 32 cases were presented sequentially within a single session for each model, which may have introduced context-dependent effects whereby earlier cases influenced the classification of later cases. Although this sequential, single-session design was adopted to mirror the conditions under which human raters performed their assessments, future studies could evaluate the impact of independent sessions or randomized case order on LLM performance. In addition, model behavior may change with version updates, and performance may differ with prompting strategies.

## Conclusions

In this pilot cohort of cardiovascular surgical patients, ASA-PS classifications generated by GPT-5.2 and Gemini 3 variants demonstrated moderate overall agreement when evaluated alongside residents and specialists, but good agreement with board-certified cardiovascular anesthesiologists and higher exact-match rates with expert consensus than residents. These findings support further multi-center validation and prospective evaluation of LLMs as adjunctive tools for ASA-PS assessment and as potential supports for anesthesiology training.

## Supplementary Information

Supplementary Material 1.

Supplementary Material 2.

Supplementary Material 3.

## Data Availability

The datasets used and/or analyzed during the current study are available from the corresponding author on reasonable request.

## Abbreviations

AI: Artificial intelligence
ASA-PS: American Society of Anesthesiologists Physical Status
ICC: Intraclass correlation coefficients
LLMs: Large language models
