# Supplementary material for: Evaluation of the reliability of large language models for ASA-PS classification in cardiovascular surgery: a pilot study
Source: JA Clin Rep. 2026 Apr 15;12:24. doi: 10.1186/s40981-026-00858-4 (PMC13194820; doi:10.1186/s40981-026-00858-4)
Supplement: Supplementary file 1 — Supplementary Material 1. [file 40981_2026_858_MOESM1_ESM.docx]

**Case1**

**Age:** 70 years

**Sex:** Male

**BMI:** 29

**NYHA:** 3

**Surgical Procedure:** Aortic Valve Replacement, Ascending Aortic Replacement

**Present Illness:**

The patient presented with exertional dyspnea. Coronary artery angiography showed no significant stenosis. Severe aortic valve stenosis and ascending aortic dilatation were noted, leading to the decision for surgery.

**Social History:** ex-smoker

**Past Medical History & Comorbidities:**

• Hypertension (HT)

• Obstructive Sleep Apnea Syndrome (OSAS) (on CPAP)

• Hyperlipidemia (HL)

**Laboratory and Examination Findings:**

• **Blood tests:** Hb 14.3 g/dL, Platelet count 153,000/μL, eGFR 60 mL/min/1.73㎡

• **ECG:** Sinus rhythm, 1st-degree AV block

• **Transesophageal Echocardiography (TEE):** EF 58%, Severe Aortic Stenosis, Ascending Aortic Dilatation (43 mm)

• **Pulmonary Function Test:** VC 4.52 L (105%), FEV1.0% 89%
