# Supplementary material for: Evaluation of the reliability of large language models for ASA-PS classification in cardiovascular surgery: a pilot study
Source: JA Clin Rep. 2026 Apr 15;12:24. doi: 10.1186/s40981-026-00858-4 (PMC13194820; doi:10.1186/s40981-026-00858-4)
Supplement: Supplementary file 2 — Supplementary Material 2. [file 40981_2026_858_MOESM2_ESM.docx]

**Prompt**

I will now present 32 cases. Please evaluate the ASA-PS classification for each case.

**Answer of Gemini**

I'm ready to help with that. The **ASA Physical Status (ASA-PS) Classification System** is a vital tool for assessing a patient's pre-operative physical state, and it can sometimes be a bit subjective depending on the severity of their systemic diseases.

To ensure we get these as accurate as possible, please provide the details for each case. For the most precise classification, it's helpful if you include:

- The patient's **age**.
- Any **chronic conditions** (and whether they are well-controlled or poorly controlled).
- Recent **acute events** (like a recent MI or CVA).
- Relevant **lifestyle factors** (like smoking status or pregnancy).

Please go ahead and list the 32 cases, and I will evaluate them for you. Would you like me to provide a brief rationale for the classification of each case as we go through them?

**Prompt**

Case1

Age: 70 years

Sex: Male

BMI: 29

NYHA: 3

Surgical Procedure: Aortic Valve Replacement, Ascending Aortic Replacement

Present Illness:

The patient presented with exertional dyspnea. Coronary artery angiography showed no significant stenosis. Severe aortic valve stenosis and ascending aortic dilatation were noted, leading to the decision for surgery.

Social History: ex-smoker

Past Medical History & Comorbidities:

• Hypertension (HT)

• Obstructive Sleep Apnea Syndrome (OSAS) (on CPAP)

• Hyperlipidemia (HL)

Laboratory and Examination Findings:

• Blood tests: Hb 14.3 g/dL, Platelet count 153,000/μL, eGFR 60 mL/min/1.73㎡

• ECG: Sinus rhythm, 1st-degree AV block

• Transesophageal Echocardiography (TEE): EF 58%, Severe Aortic Stenosis, Ascending Aortic Dilatation (43 mm)

• Pulmonary Function Test: VC 4.52 L (105%), FEV1.0% 89%

**Answer of Gemini 3 Fast**

Based on the clinical details provided, here is the evaluation for Case 1:

**Case 1 Evaluation: ASA-PS Class 3**

**Rationale**

The patient is classified as **ASA 3** (a patient with severe systemic disease). While several factors contribute to this, the primary driver is the symptomatic cardiovascular disease:

- **NYHA Class 3 Heart Failure Symptoms:** The patient is experiencing exertional dyspnea, indicating that their physical activity is significantly limited by their cardiac condition.
- **Severe Aortic Stenosis (AS):** Symptomatic valvular heart disease is considered a severe systemic disease. Even though the Ejection Fraction (EF) is preserved at **58%**, the presence of severe stenosis with functional impairment (NYHA 3) warrants an ASA 3 classification.
- **Comorbidities:** The patient has a cluster of conditions—**Hypertension**, **Hyperlipidemia**, and **Obstructive Sleep Apnea (OSAS)** requiring CPAP—which, while likely managed, contribute to the overall systemic burden.
- **Renal Function:** An **eGFR of 60** sits right at the threshold of Stage 2/3 Chronic Kidney Disease, further supporting a Class 3 designation.

**Summary Table**

| **Factor** | **Clinical Data** | **Impact on ASA** |
| --- | --- | --- |
| **Cardiac Functional Status** | NYHA Class 3 | **Severe systemic disease** |
| **Primary Diagnosis** | Severe AS / Ascending Aortic Dilation | High-risk surgical pathology |
| **Respiratory** | OSAS on CPAP | Controlled, but increases perioperative risk |
| **Renal** | eGFR 60 mL/min/1.73㎡ | Mildly decreased function |

Please provide the details for **Case 2** whenever you are ready! Would you like me to continue with this same level of detail for the remaining cases?
