# Supplementary material for: Evaluation of the reliability of large language models for ASA-PS classification in cardiovascular surgery: a pilot study
Source: JA Clin Rep. 2026 Apr 15;12:24. doi: 10.1186/s40981-026-00858-4 (PMC13194820; doi:10.1186/s40981-026-00858-4)
Supplement: Supplementary file 3 — Supplementary Material 3. [file 40981_2026_858_MOESM3_ESM.docx]

**Supplemental Table 1. Pairwise quadratic weighted kappa between LLMs and human raters**

| **LLMs vs Board-Certified Cardiovascular Anesthesiologists** | | |  | |
| --- | --- | --- | --- | --- |
| **LLM** | **vs Specialist A** | **Interpretation** | **vs Specialist B** | **Interpretation** |
| GPT-5.2 Instant | 0.52 (0.23–0.73) | Moderate | 0.64 (0.40–0.81) | Substantial |
| GPT-5.2 Thinking | 0.71 (0.42–0.89) | Substantial | 0.47 (0.17–0.69) | Moderate |
| Gemini 3 Fast | 0.74 (0.50–0.91) | Substantial | 0.46 (0.20–0.66) | Moderate |
| Gemini 3 High Thinking | 0.69 (0.41–0.87) | Substantial | 0.49 (0.23–0.69) | Moderate |
| **LLMs vs Anesthesiology Residents** | | |  | |
| **LLM** | **vs Resident A** | **Interpretation** | **vs Resident B** | **Interpretation** |
| GPT-5.2 Instant | 0.14 (−0.07–0.35) | Slight | 0.35 (0.13–0.56) | Fair |
| GPT-5.2 Thinking | 0.27 (0.00–0.50) | Fair | 0.68 (0.43–0.86) | Substantial |
| Gemini 3 Fast | 0.43 (0.15–0.61) | Moderate | 0.61 (0.33–0.82) | Substantial |
| Gemini 3 High Thinking | 0.37 (0.03–0.59) | Fair | 0.73 (0.44–0.90) | Substantial |

Values represent quadratic weighted kappa coefficients with 95% confidence intervals.

Interpretation (Landis & Koch): ≤0.20 = slight; 0.21–0.40 = fair; 0.41–0.60 = moderate; 0.61–0.80 = substantial; ≥0.81 = almost perfect agreement.

※Abbreviation: LLM, large language model.
